# A Novel *MAG* Variant Causes Hereditary Spastic Paraplegia in a Consanguineous Pakistani Family

**DOI:** 10.3390/genes15091203

**Published:** 2024-09-13

**Authors:** Rabia Akram, Haseeb Anwar, Humaira Muzaffar, Valentina Turchetti, Tracy Lau, Barbara Vona, Ehtisham Ul Haq Makhdoom, Javed Iqbal, Shahid Mahmood Baig, Ghulam Hussain, Stephanie Efthymiou, Henry Houlden

**Affiliations:** 1 Neurochemicalbiology and Genetics Laboratory (NGL), Department of Physiology, Faculty of Life Sciences, Government College University, Faisalabad 38000, Pakistan; rabiaakram26@gcuf.edu.pk (R.A.); drhaseebanwar@gcuf.edu.pk (H.A.); drhumairamuzaffar@gcuf.edu.pk (H.M.); shaamisaahib@gmail.com (E.U.H.M.); 2 Department of Neuromuscular Disorders, UCL Queen Square Institute of Neurology, London WC1N 3BG, UK; v.turchetti@ucl.ac.uk (V.T.); tracy.lau@ucl.ac.uk (T.L.); h.houlden@ucl.ac.uk (H.H.); 3 Institute of Human Genetics, University Medical Center Göttingen, 37073 Göttingen, Germany; barbara.vona@med.uni-goettingen.de; 4 Institute for Auditory Neuroscience and InnerEarLab, University Medical Center Göttingen, 37075 Göttingen, Germany; 5 Human Molecular Genetics Laboratory, Health Biotechnology Division, National Institute for Biotechnology and Genetic Engineering (NIBGE) College, Faisalabad 38000, Pakistan; shahid_baig2002@yahoo.com; 6 Department of Neurology, Allied Hospital, Faisalabad Medical University, Faisalabad 38000, Pakistan; drjavedneurologist@gmail.com; 7 Department of Biological and Biomedical Sciences, Aga Khan University, Stadium Road, Karachi 74000, Pakistan

**Keywords:** cerebellar ataxia, spastic paraplegia, Pakistan, *MAG*, SPG75

## Abstract

**Background and objectives:** Hereditary spastic paraplegia (HSP) is characterized by unsteady gait, motor incoordination, speech impairment, abnormal eye movement, progressive spasticity and lower limb weakness. Spastic paraplegia 75 (SPG75) results from a mutation in the gene that encodes myelin associated glycoprotein (MAG). Only a limited number of *MAG* variants associated with SPG75 in families of European, Middle Eastern, North African, Turkish and Palestinian ancestry have been documented so far. This study aims to provide further insight into the clinical and molecular manifestations of HSP. **Methods:** Using whole-exome sequencing, we investigated a consanguineous Pakistani family where three individuals presented with clinical signs of HSP. Sanger sequencing was used to carry out segregation analysis on available family members, and a minigene splicing assay was utilized to evaluate the effect of the splicing variant. **Results:** We identified a novel homozygous pathogenic splice donor variant in *MAG* (c.46 + 1G > T) associated with SPG75. RNA analysis revealed exon skipping that resulted in the loss of a start codon for ENST00000361922.8 isoform. Affected individuals exhibited variable combinations of nystagmus, developmental delay, cognitive impairments, spasticity, dysarthria, delayed gait and ataxia. The proband displayed a quadrupedal stride, and his siblings experienced frequent falls and ataxic gait as one of the prominent features that have not been previously reported in SPG75. **Conclusions:** Thus, the present study presents an uncommon manifestation of SPG75, the first from the Pakistani population, and broadens the spectrum of *MAG* variants.

## 1. Introduction

Hereditary spastic paraplegias (HSPs) are primarily defined by increasing spasticity, impaired gait, and lower limb weakness brought on by distal degeneration of corticospinal tract neurons [1]. Clinically, HSPs exhibit a high degree of diversity in both pure and complex forms. They can manifest globally at any age, with adult onset, pediatric, and congenital presentations observed. Various forms of HSPs are classified according to chromosomal locus, mode of inheritance, and specific causative genes or mutations [2]. An autosomal recessive neurodegenerative disorder known as spastic paraplegia type 75 (SPG75, OMIM*616680) is distinguished by the early onset of spastic paraplegia. This condition is caused by a mutation in the gene that encodes myelin-associated glycoprotein (MAG, OMIM*159460), which is located on chromosome 19q13 [3].

MAG, a well-known protein, is a member of the lectin superfamily SIGLEC and can bind sialic acid, which is involved in myelination, glial–axon maintenance and axon–glial interactions during nerve regeneration [4]. It is also implicated in axonal regeneration, neurite outgrowth inhibition [5] and neuroprotection from axonal damage [6]. Only a limited number of *MAG* variants associated with SPG75 have been reported so far [3,4,7,8,9,10,11]. The main clinical features presented in these studies were ataxia, intellectual disability, dystonia, amyotrophy, epilepsy, oculomotor delay, and peripheral neuropathy. 

Herein, the current study investigated a consanguineous Pakistani family presenting with an HSP-associated phenotype through whole-exome sequencing. We identified a first-ever occurrence of a homozygous splice donor variant in *MAG* (c.46 + 1G > T) in three affected family members who exhibited SPG75. Quadrupedal gait in the proband and frequent falls, ataxic wide-based gait and strabismus in the siblings were the novel manifestations found in our study. Thus, this investigation expanded the mutation spectrum of *MAG* variants related to HSPs. 

## 2. Materials and Methods

### 2.1. Recruitment of Family

The consanguineous family was examined at the Department of Neurology, Allied Hospital Faisalabad, in 2022 as part of research project aimed at identifying causative genes associated with spastic paraplegia (Faisalabad, Pakistan). In their native language, the family was informed about the study’s purpose and nature. The senior neurologist evaluated the patients and obtained informed consent. This investigation was authorized by the Government College University Faisalabad (IRB-3826) and followed the guidelines from the Declaration of Helsinki. We collected blood samples from the hospital for genetic studies. We searched Google scholar and PubMed to identify previously published studies with SPG75 using the keywords “MAG, hereditary spastic paraplegia, autosomal-recessive, and spastic paraplegia 75”.

### 2.2. Genetic Analysis

**Whole exome sequencing:** Genomic DNA was extracted using a method that included proteinase digestion, sucrose lysis, and salting out from the peripheral blood of affected individuals (V.1, V.2, V.3), their siblings (V.4, V.5), and the parent who was accessible (IV:12, the mother). Using the Agilent V4 enrichment kit (Santa Clara, CA, USA), the genomic DNA of the proband was prepared for whole-exome sequencing. Paired-end reads were obtained using an Illumina HiSeq 2500 sequencer with 100× coverage (Macrogen, Seoul, Republic of Korea). 

The wANNOVAR program was utilized to annotate exome data. Given the families were consanguineous and the inheritance pattern was recessive, only homozygous variants were taken into account. Allele frequencies greater than or equal to 0.01 in any publicly accessible database (dbSNP, gnomAD, ExAC, and the 1000 Genome Project) were used to filter out variants. Variants expected to affect splicing and those located within exons were taken into consideration. From the wANNOVAR analysis file, the pathogenicity scores predicted by Combined Annotation Dependent Depletion (CADD), FATHMM, and PROVEAN were obtained [12]. Additionally, we excluded variants with CADD [13,14] scores less than 10. We employed the American College of Medical Genetics and Genomics (ACMG) variant categorization approach to predict pathogenic and likely pathogenic variants [15]. In silico pathogenicity prediction tools like PolyPhen-2, SIFT, and MutationTaster were employed for further confirmation. To eliminate any artifacts and false positives, candidate variants were visually examined using the Integrative Genomics Viewer (IGV) [16]. SpliceSiteFinder-like, NNSplice, MaxEntScan, RESCUE-ESE, ESEfinder, and GeneSplicer integrated in Alamut Visual Plus v1.6.1 (Sophia Genetics, Bidart, France), as well as SpliceAI Visual were employed for the computational evaluation of variants for possible splicing effects [17].

**Prediction of translation initiation sites:** The program Predict TIS was used to explore alternative in-frame translation start sites to evaluate the effect of exon skipping as a result of an alternative splicing event caused by the mutation (c.46 + 1G > T) [18]. This tool separates ATG and near-cognate translation starting codon for translation initiation. Kozak similarity score produced color-coded ATG predictions for the wild-type MAG messenger RNA sequence (ENST00000361922.8). The scale used to calculate the scores is from less than 0.5 to higher than 0.8. The codes are blue (scores < 0.5), teal (≥0.5 and <0.6), green (≥0.6 and <0.7), orange (≥0.7 and <0.8), and red (≥0.8). 

**Sanger sequencing:** BigDye Terminator was used to prepare samples for Sanger sequencing. The specific primers (Table S1) of the variant-containing areas were designed for PCR amplification. All available family samples underwent segregation analysis, and the Lasergene program was used to interpret sequencing data (DNASTAR Inc., Madison, WI, USA). A group of individuals with shared genetic ancestry provided 100 control chromosomes to determine the prevalence of the recently discovered pathogenic variants [12].

**In vitro splice assay for functional analysis of the c.46 + 1G > T variant:** The *MAG* c.46 + 1G > T variant was computationally analyzed to assess potential aberrant splicing [19]. Genomic DNA amplification was used for an in vitro experiment targeting exon 3 along with flanking UTR and intronic regions to functionally evaluate the anticipated impact of the *MAG* c.46 + 1G > T variant [20,21]. Primer sequences are shown in Table S2. Before ligating the pSPL3 exon trapping vector with the amplified target sequence (containing exons A and B of the linearized vector) and transforming it into DH5α-competent cells (NEB 5α, New England Biolabs, Frankfurt, Germany), the PCR amplicon and the pSPL3 exon trapping vector were purified and subjected to restriction enzyme digestion (*Xho*I, *Bam*HI). The cells were plated for overnight incubation. A colony PCR screening approach was employed, incorporating an SD6 forward primer and an exon-specific reverse primer, to screen colonies based on size, with Sanger sequencing validating the inserts. At a density of 2 × 10^5^ cells per millilitre, HEK 293T cells (ATCC, Manassas, VA, USA) were transfected with sequence-confirmed vectors containing both mutant and wild-type sequences. A transient transfection was conducted using 6 µL of FuGENE 6 Transfection Reagent on 2 µg of the corresponding pSPL3 vectors (Promega, Walldorf, Germany). Transfection-negative and empty vector samples were used as controls. The transfected cells were harvested 24 h post-transfection. An miRNeasy Mini Kit was used to prepare total RNA (Qiagen, Hilden, Germany). A High Capacity cDNA Reverse Transcription Kit (Applied Biosystems, ThermoFisher Scientific, Waltham, MA, USA) was employed to reverse transcribe RNA (2 µg) by following the manufacturer’s protocol. cDNA was amplified by PCR using vector-specific SD6 forward and SA2 reverse primers. Sanger sequencing was performed once the amplified fragments were visualized on a 1% agarose gel (Sigma, Darmstadt, Germany). 

## 3. Results

**Figure 1:**
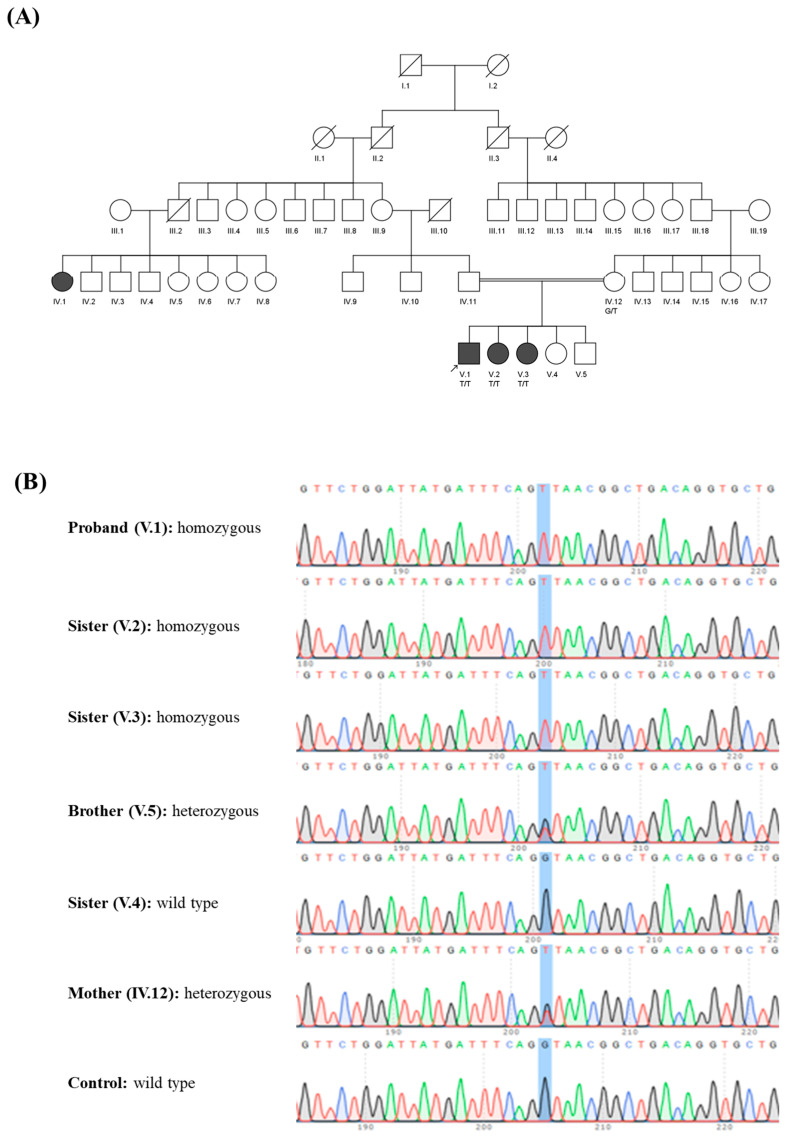
(**A**) Five-generation pedigree of the family showing three affected siblings. All affected individuals were homozygous (G > T) while the mother was heterozygous (Proband: V.1; Affected sister: V.2; Affected sister: V.3). The sample for IV.1 was unavailable for further study. (**B**) Sequence chromatograms of *MAG* showing a likely pathogenic c.46 + 1G > T variant.

**Clinical details:** Three affected members of the Pakistani family (Figure 1A) presented with mild dysarthria, delayed milestones, coordination impairment, inability to walk, nystagmus, muscle weakness, tremors, body shivering, frequent falls, strabismus and intellectual disability (Table 1). The gait ataxia scaled to quadrupedal gait in the proband. One brother and sister in the pedigree were healthy. At the time of the assessment, the patients’ ages ranged from 8 to 12 years, although symptoms were noticed starting from one year of age. The affected individuals were vaginally delivered normally and at term. The mother had uncomplicated pregnancies and deliveries. Nevertheless, the woman had sickness throughout her pregnancy. 

The proband (V.1) was still dependent on self-care and feeding. Standing and walking were never achieved. He exhibited quadrupedal gait, dysarthria, and head and hand tremors. All of the affected individuals had moderate to severe abnormalities in their speech, posture, and gait. There were tremors, dysdiadochokinesia, and moderate to severe dysmetria. Every patient had nystagmus and strabismus, according to an ophthalmological examination. V.2 exhibited mild dysarthria, frequent falls, developmental delays, hand tremors, and gait delays. She also showed low body weight and slow growth. She had never attended school. 

Interestingly, V.3 was able to attend a special education school and could walk independently. She had a short temper and became upset over minor issues, causing her body to tremble. Despite experiencing slight dysarthria, she spoke normally. She fell quickly even after a short stroll. The patient was able to eat and drink independently, and her height and weight were within normal ranges. However, her movements continued to be clumsy and sluggish. Additionally, we found no neurological abnormalities in her parents. 

**Genetic analyses:** A novel likely pathogenic homozygous splice donor variation (c.46 + 1G > T) in the *MAG* (GenBank: NM 002361.4) was identified by exome sequencing analysis. The variant had a CADD score of 29.1 and was absent in the gnomAD database (CADD model GRCh38-v1.6). This variant was classified by ACMG guidelines for variant interpretation as likely pathogenic using criteria PM2 supporting and PVS1. An in silico study predicted this variant to be deleterious (DANN score = 0.99; Mutation Taster score = 1). We postulated that an alternate translation start point is employed rather than a total loss of *MAG* expression based on Kozak consensus sequence. Searching the human MAG mRNA sequence revealed 11 Kozak consensus sequence predictions. Position 1090 in exon 5 (score of 0.56) corresponds to c.478 and is the closest in-frame projected Kozak consensus sequence to the original start codon (position 159 with a value of 0.64). Utilizing this start codon would result in a deletion affecting all of exons 3, 4, and part of exon 5, truncating the first 159 amino acids of the MAG protein. Sanger sequencing confirmed an autosomal recessive mode of inheritance and demonstrated segregation of the distinct pathogenic variant with the phenotype, with affected individuals being homozygous and the mother carrying a heterozygous mutation (Figure 1B). 

**Figure 2:**
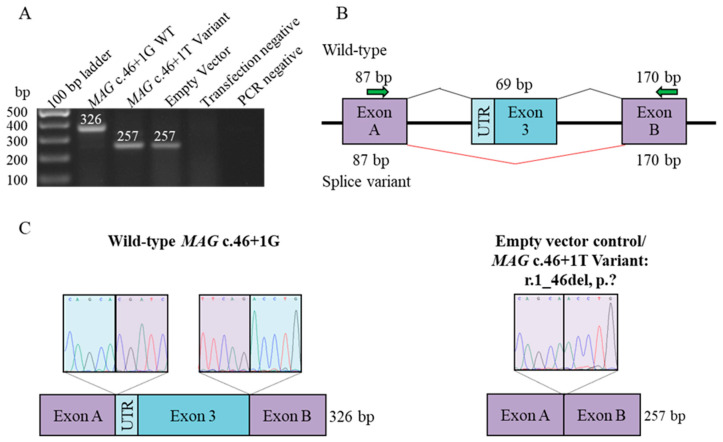
RNA functional studies of the *MAG* c.46 + 1G > T variants. (**A**) RT-PCR amplicons for the *MAG* c.46 + 1G wild-type, c.46 + 1T mutant, and empty pSPL3 vector were separated by gel electrophoresis. The PCR and transfection negative controls performed as expected. (**B**) The in vitro splice assay’s vector construct displays the variant-containing (lower splice profile) and wild-type (upper splice profile) amplicons that are placed between pSPL3 vector’s exons A and B. Each variant’s splicing schematic is displayed below. The c.46 + 1G > A variant causes skipping of exon 3, resulting in a deletion of 46 bp of coding exon 3 (r.1_46del), p.?, including the start codon of the ENST00000361922.8/NM_080600.3 isoforms. (**C**) Sequencing of the exon–exon junctions for the wild-type (left) and variant, appearing as the empty vector control (right).

In silico splice prediction of the *MAG* c.46 + 1G > T variant indicates the activation of a cryptic splice donor site in the first coding exon (Exon/Intron 3 out of 11 coding exons (12 total), whereby the first coding exon is 3 (Figure S1). SpliceAI Visual prediction revealed an alternative transcript where a splice acceptor site of a UTR is reduced. The variant was predicted to abolish the native splice donor site. RNA analysis of the amplicons containing this variant showed evidence of exon skipping (Figure 2A–C) and suggested that this would result in the loss of a start codon for ENST00000361922.8/NM_080600.3 isoforms.

## 4. Discussion

Based on clinical evidence, HSP exhibits genetic heterogeneity and has been categorized into various subtypes. To date, numerous causative genes of HSP have been investigated, including those with autosomal recessive, autosomal dominant, mitochondrial, and X-linked inheritance patterns. The underlying pathophysiological mechanisms associated with HSP are becoming clearer as more causative genes have been identified in recent years. HSP-linked genes encode proteins that are crucial for several physiological processes, such as myelination, axonal transport, lipid metabolism, intracellular trafficking, and mitochondrial function [22,23]. 

The current investigation identified a new likely pathogenic splice donor variation in homozygosity in *MAG* (c.46 + 1G > T) in a consanguineous Pakistani family with three affected individuals. *MAG* emerged as the primary candidate gene to cause HSP and frequently overlaps with hereditary cerebellar ataxia (HCA). The knowledge that HSP and HCA share many clinical and pathophysiological aspects has contributed to identifying many genes responsible for both cerebellar and pyramidal symptoms [24,25]. Furthermore, several individuals without pyramidal symptoms had a major cerebellar ataxia phenotype caused by genes typically linked to HSP, such as spastic paraplegia 7 (SPG7; OMIM*607259) [26,27]. 

**Figure 3:**
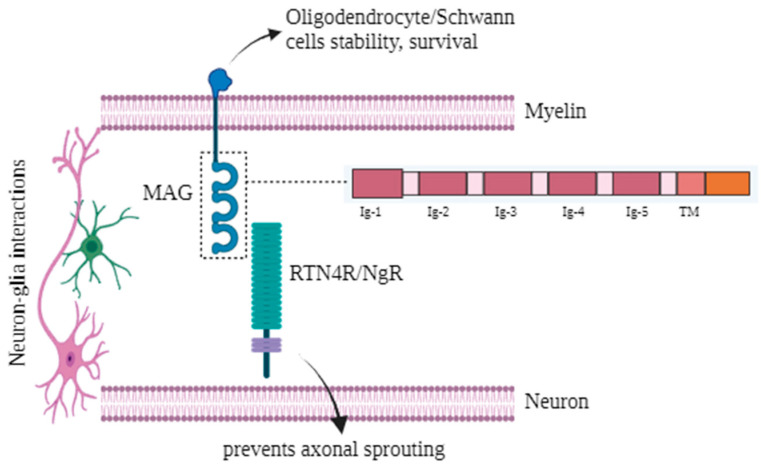
MAG interacts with RTN4R/NgR and prevents axonal sprouting. MAG contains five Ig domains (1–5) and a intramembrane segment. MAG: Myelin associated glycoprotein; RTN4R: reticulon-4 receptor; NgR: Nogo Receptor; Ig: Immunoglobulin domain; Transmembrane domain: TM. Created with BioRender.com, accessed on 6 September 2024.

MAG is a sialic acid binding SIGLEC protein. The extracellular segment of MAG comprises five immunoglobulin like domains (disulphide-bonded), a cytoplasmic region, and a single intramembrane segment (Figure 3). The first three immunoglobulin domains of MAG interact with the reticulon-4 receptor (RTN4R) and homo-dimerize via the fourth and fifth immunoglobulin domains. MAG is a myelin constituent present in Schwann cells and oligodendrocytes. It is crucial for the development and maintenance of myelinated axons. Additionally, it serves as a cell-recognition molecule that plays a role in the connections between neurons and glia, regulating axon development and the regeneration of nerve tissue. It inhibits axonal sprouting and regeneration, affecting axonal viability and cytoskeletal architecture [28,29,30]. 

Taking into account the present series of new and published cases, the patients in our study exhibited characteristics similar to those of previously reported individuals affected by SPG75, including developmental delay, ataxia, and significant motor dysfunction. The features observed in all patients associated with *MAG* mutations included ataxia (20/20), developmental delay (12/20), dysarthria (12/20), nystagmus (16/20), spasticity (15/20), pyramidal signs (13/20), hyporeflexia (12/20), delayed gait (10/20), and neuropathy (10/20) (Table 1).

Quadrupedal gait is reported for the first time in the proband with SPG75. Wide based gait and ataxia were the prominent clinical features presented by V.2 and V.3. In silico prediction revealed that the variant in *MAG* is located at intron 3 of 11 (splicing-ACMG, splicing, intronic) and it leads to loss of a start codon for the ENST00000361922.8 isoform.

*MAG* has been shown to have missense, nonsense, frameshift, and splicing variants (Table 1). In 2014, Novarino and colleagues [7] reported *MAG* as the candidate gene for the first time segregating with complex spastic disorder in a single consanguineous family. Later on, Lossos et al. studied in vitro functional expression and revealed that missense variant impacted MAG’s glycosylation and posttranslational modification. It also resulted in the retention of the mutant protein in the endoplasmic reticulum, where it was degraded by proteases. The clinical characteristics were compatible with anomalies in the central and peripheral nerve systems [3].

Afterwards, homozygous and heterozygous variants in *MAG* were reported in individuals from North African [8], European, and Middle Eastern [10], European [9], and Turkish [11] origins, presenting varying combinations of psychomotor delay, abnormalities in eye movements, oculomotor apraxia, ataxia, spasticity, nystagmus, dystonia, dysfunction of the pyramidal tract, and neuropathic symptoms. 

Thus far, only 17 patients have been reported from different ethnic groups. This study raised the total number of SPG75-implicated variants in *MAG* to twelve. This work is noteworthy since it identifies a novel variant and it is the first case of *MAG*-associated disorder found in Pakistani ethnicity. This report highlighted the need to further investigate and explore hidden variants to address the geographic spectrum of genetic disorders in developing countries like Pakistan. This study might assist in identifying families for cascade testing to lower the illness burden through genetic testing, and it would help professionals diagnose patients.

## Figures and Tables

**Table 1:** A summary of the clinical and genetic information for HSP individuals.

Present Study				Previous Studies							
Patient Identifier	V.1	V.2	V.3	[7] 1226-IV-3	[7] 1226-IV-4	[3] III-2	[3] III-3	[3] III-5	[4] II.2	[4] II.5	[8]
**Transcript**	NM_080600.3	NM_080600.3	NM_080600.3	NM_002361.3	NM_002361.3	NM_002361.3	NM_002361.3	NM_002361.3	NM_002361.3	NM_002361.3	NM_002361.3
**Mutation**	c.46 + 1G > T	c.46 + 1G > T	c.46 + 1G > T	c.1288T > G (p.Cys430Gly)	c.1288T > G (p.Cys430Gly)	c.399C > G (p.Ser133Arg)	c.399C > G (p.Ser133Arg)	c.399C > G (p.Ser133Arg)	c.353G > A (p.Arg118His)	c.353G > A (p.Arg118His)	c.452C > T (p.Ala151Val); c.1117A > C (p.Ser373Arg)
**Zygosity**	Hom	Hom	Hom	Hom	Hom	Hom	Hom	Hom	Hom	Hom	Comp Het
**Consequence**	Splice donor	Splice donor	Splice donor	Missense	Missense	Missense	Missense	Missense	Missense	Missense	-
**Sex**	M	F	F	F	F	M	M	F	M	M	F
**Age of onset (yr)**	1	1	1	N/A	N/A	N/A	N/A	N/A	N/A	N/A	N/A
**Age of last examination (yr)**	17	15	13	12	11	28	26	21	54	48	10
**Ethnic background**	Pakistan	Pakistan	Pakistan	N/A	N/A	Middle Eastern	Middle Eastern (Palestinian Arab)	Middle Eastern	N/A	N/A	African
**Consanguinity**	+	+	+	+	+	+	+	+	+	+	-
**Developmental delay**	+	+	+	N/A	N/A	+	+	+	+	N/A	+
**Hypotonia**	N/A	N/A	N/A	N/A	N/A	+	+	+	N/A	N/A	N/A
**Intellectual disability**	+	+	+	N/A	N/A	+	+	+	+	+	+
**Cognitive impairment**	+	+	+	+	+	+	+	+	+	+	+
**Dysarthria**	+	+	+	-	-	+	+	+	+	N/A	N/A
**Nystagmus**	+	+	+	+	+	+	+	+	+	+	N/A
**Other ophthalmologic findings**	+	+	+	-	-	+	+	+	+	+	+
**Spasticity**	+	+	+	+	+	+	+	+	+	-	+
**Pyramidal signs**	+	+	+	+	+	+	+	+	+	+	+
**Ataxic signs**	+	+	+	+	+	+	+	+	+	+	+
**Dystonia**	N/A	N/A	N/A	-	-	-	-	-	-	-	-
**Hypo-/areflexia**	+	+	+	-	-	+	+	+	+	+	+
**Vibratory sense deficit**	-	-	-	+	+	+	+	+	+	+	N/A
**Muscular atrophy**	N/A	N/A	N/A	+	+	+	+	+	N/A	N/A	+
**Delay of gait**	NA (Quadrupedal gait)	+	+	N/A	N/A	+	+	+	-	+	-
**Neuropathy**	N/A	N/A	N/A	N/A	N/A	+	+	+	+	+	+
**Support for walking**	N/A	-	-	N/A	N/A	+	+	+	N/A	N/A	+
**Motor Deficit**	+	+	+	+	+	+	+	+	+	+	+
**Scoliosis**	-	-	-	N/A	N/A	-	-	-	N/A	N/A	N/A
**limb dysmetria**	+	+	+	N/A	N/A	+	+	+	+	+	+
**Intention tremor**	+	+	+	N/A	N/A	-	-	-	N/A	N/A	N/A
**Patient Identifier**	[10]			[10]	[10]	[10]	[9]	[9]	[9]	[11]	[11]
	**A-II-1**			**B-II-1**	**C-II-1**	**D-II-1**	**II.1**	**II.2**	**II.3**	**P13**	**P14**
**Transcript**	NM_002361.3			NM_002361.3	NM_002361.3	NM_002361.3	-	-	NP_002352.1	NM_002361	NM_002361
**Mutation**	c.1126C > T (p.Gln376)			c.517_521dup (p.Trp174)	c.1522C > T (p.Arg508)	c.693C > A (p.Tyr231); c.980G > A (p.Trp327)	c.124T > C(p.Cys42Arg)	c.124T > C (p.Cys42Arg)	c.124T > C(p.Cys42Arg)	c.475T > G (p.Cys159Gly)	c.475 T > G (p.Cys159Gly)
**Zygosity**	Hom			Hom	Hom	Comp Het	Hom	Hom	Hom	Hom	Hom
**Consequence**	Nonsense			Frameshift	Nonsense	Nonsense	Missense	Missense	Missense	N/A	N/A
**Sex**	M			F	F	F	M	M	F	M	F
**Age of onset (yr)**	Early infancy			N/A	N/A	N/A	1	1	1	1-2 month	1-2 month
**Age of last examination (yr)**	35			21	2.5	12	59	56	54	10	3
**Ethnic background**	European			European	Middle eastern	European	Portuguese	Portuguese	Portuguese	Turkish	Turkish
**Consanguinity**	-			+	+	-	+	+	+	+	+
**Developmental delay**	+			+	+	+	N/A	N/A	N/A	-	-
**Hypotonia**	+			-	+	+	N/A	N/A	N/A	+	+
**Intellectual disability**	+			+	+	+	N/A	N/A	N/A	-	-
**Cognitive impairment**	-			+	-	-	-	-	-	N/A	N/A
**Dysarthria**	+			+	-	+	N/A	N/A	N/A	+	+
**Nystagmus**	+			+	+	+	N/A	N/A	N/A	+	+
**Other ophthalmologic findings**	+			-	-	+	+ (oculomotor apraxia)	++	++	-	-
**Spasticity**	+			+	-	+	-	-	-	+ (in lower limbs only)	+ (in lower limbs only)
**Pyramidal signs**	+			+	-	-	-	-	-	N/A	N/A
**Ataxic signs**	+			+	+	+	+ (First sign)	+ (First sign)	+ (First sign)	++ (12 month)	++ (12 month)
**Dystonia**	+			+	-	-	-	-	-		
**Hypo-/areflexia**	+			-	+	+	-	-	-	N/A	N/A
**Vibratory sense deficit**	+			-	-	+	N/A	N/A	N/A	N/A	N/A
**Muscular atrophy**	+			-	-	-	N/A	N/A	N/A	N/A	N/A
**Delay of gait**	N/A			N/A	Wide base gait	N/A	+	+	+	N/A	N/A
**Neuropathy**	+			-	N/A	-	+ (Prominent)	+ (Prominent)	+ (Prominent)	N/A	N/A
**Support for walking**	N/A			+	N/A	+	+			N/A	N/A
**Motor Deficit**	+			+	+	+	+++	+++	+++	-	-
**Scoliosis**	+			N/A	N/A	N/A	-	-	-	N/A	N/A
**limb dysmetria**	N/A			N/A	N/A	N/A	+	+	+	N/A	N/A
**Intention tremor**	N/A			N/A	N/A	N/A	N/A	+	+	N/A	N/A

+: Present; -: absent; ++: moderate; +++: severe; M: Male; F: Female; Homozygous: Hom; Compound Heterozygous: Comp. Het; N/A: Not Available.

## Data Availability

The data from this study are provided as Supplementary Materials. The sharing of family pictures and videos is restricted due to ethical concerns.

## Supplementary Materials

The following supporting information can be downloaded at: https://www.mdpi.com/article/10.3390/genes15091203/s1, Figure S1: In silico prediction of the *MAG*: c.46 + 1G > T variant; Table S1: Oligos designed for Sanger sequencing; Table S2: List of primers used for the minigene assay; Table S3: Filtered variant data.
