# Supplementary material for: A Novel MAG Variant Causes Hereditary Spastic Paraplegia in a Consanguineous Pakistani Family
Source: Genes (Basel). 2024 Sep 13;15(9):1203. doi: 10.3390/genes15091203 (PMC11431006; doi:10.3390/genes15091203)
Supplement: Supplementary file 1 [file genes-15-01203-s001.zip › genes-3197984-supplementary/Supplementary file A novel MAG variant causes hereditary spastic paraplegia in a consanguineous Pakistani family.pdf]

(A)

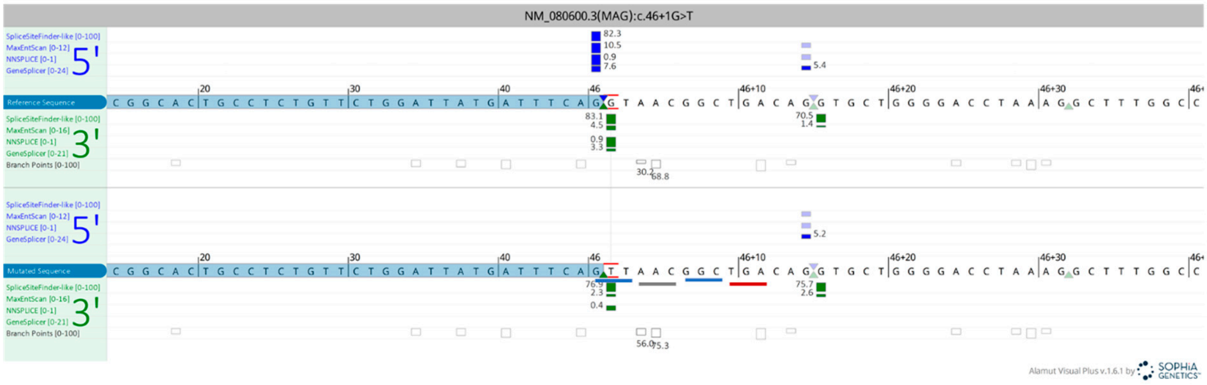

(B)

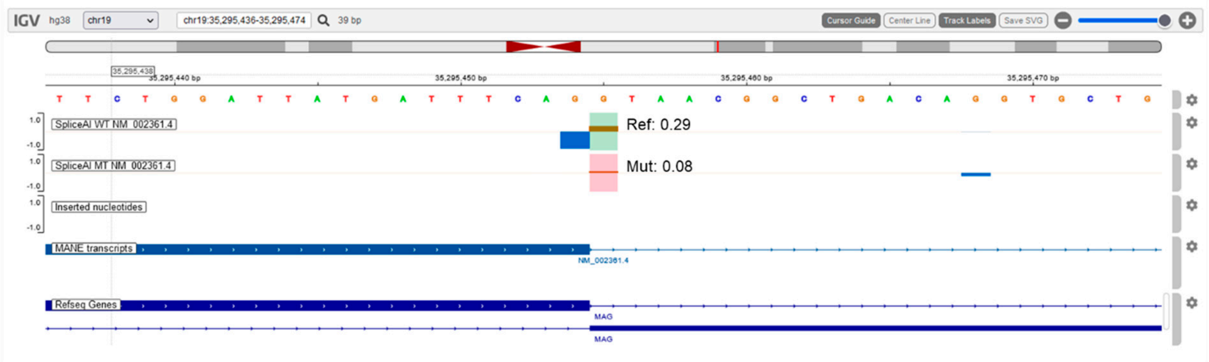

(C)

Features	Values	Descriptions
dbscSNV ADA:	1.00	Raw score 0-1, threshold ≥ 0.8 for impact
dbscSNV RF:	0.92	Raw score 0-1, threshold ≥ 0.8 for impact
spliceAI AG:	0.00 (-47)	Acceptor Gain, Δ score 0-1 (relative position in bp), thresholds ≥ 0.2|0.5|0.8 for impact
spliceAI AL:	0.21 (0)	Acceptor Loss, Δ score 0-1 (relative position in bp), thresholds ≥ 0.2|0.5|0.8 for impact
spliceAI DG:	0.32 (48)	Donor Gain, Δ score 0-1 (relative position in bp), thresholds ≥ 0.2|0.5|0.8 for impact
spliceAI DL:	0.92 (-1)	Donor Loss, Δ score 0-1 (relative position in bp), thresholds ≥ 0.2|0.5|0.8 for impact
AbSplice Max Score:	0.31 (Brain Amygdala)	AbSplice Max Tissue score, thresholds ≥ 0.01|0.05|0.2 for impact

Figure S1: *In silico* prediction of the *MAG*: c.46+1G>T variant. (**A**) *In silico* splice prediction of the wild-type (top track, red marked G) and c.46+1G>T (bottom track, red marked T). Only scores that are predicted to change are shown. (A) The *MAG* c.46+1G>T variant occurs in the first coding exon. (**B**) SpliceAI Visual prediction. The mutant base (T, bottom track) and wild-type (G, top track) are indicated by pink and green bars, respectively, to the right of the exon border. **(C)** Splice prediction scores including AbSplice, dbscSNV and Splice AI scores [score range: 0–1]. Abbreviations: Ref, reference; Mut, mutant; WT, wild-type.

Table S1: Oligos designed for Sanger sequencing

MAG**:** ENST00000361922.8:c.46+1G>T	Left primer	cgggcaatgaaacaagaccc
	Right primer	agctcatccgggaagtcaaa

Table S2. List of primers used for the minigene assay

Primer name	Primer sequence (5′-3′)	Purpose
hu_MAG_Ex3 XhoI_F	aattctcgagCGGGCAATGAAACAAGACCC	Amplification of gDNA and cloning
hu_MAG_Ex3 BamHI_R	attggatccCGAGATGGACGAGGGCATC	Amplification of gDNA and cloning
SD6 F	TCTGAGTCACCTGGACAACC	Colony PCR and RT-PCR
SA2 R	ATCTCAGTGGTATTTGTGAGC	RT-PCR
